# Characterization of the IgA response to PRRS virus in pig oral fluids

**DOI:** 10.1371/journal.pone.0229065

**Published:** 2020-03-03

**Authors:** Jessica Ruggeri, Gianluca Ferlazzo, Maria Beatrice Boniotti, Lorenzo Capucci, Flavia Guarneri, Ilaria Barbieri, Giovanni Loris Alborali, Massimo Amadori

**Affiliations:** 1 Laboratory of Animal Welfare, Clinical Chemistry and Veterinary Immunology, Istituto Zooprofilattico Sperimentale della Lombardia e dell’Emilia Romagna, Brescia, Italy; 2 Genomics Department, Istituto Zooprofilattico Sperimentale della Lombardia e dell’Emilia Romagna, Brescia, Italy; 3 Virology Department, Istituto Zooprofilattico Sperimentale della Lombardia e dell’Emilia Romagna “Bruno Ubertini” (IZSLER), Brescia, Italy; 4 Diagnostic Laboratory, Istituto Zooprofilattico Sperimentale della Lombardia e dell’Emilia Romagna, Brescia, Italy; Instituto Butantan, BRAZIL

## Abstract

Porcine Reproductive and Respiratory Syndrome (PRRS) is a complex model of host/virus relationship. Disease control measures often includes “acclimatization”, i.e. the exposure of PRRS-naïve gilts and sows to PRRSV-infected pigs and premises before the breeding period. In this respect, we had repeatedly observed an association between PRRSV-specific IgA responses in oral fluids (OF) of gilts and block of PRRSV spread. Therefore, we set out to investigate *in vitro* the inhibition of PRRSV replication by OF samples with different titers of PRRSV-specific IgA and IgG antibody, using Real-time RT PCR. PRRSV yield reduction in monocyte-derived macrophages was associated with the IgA content in OF samples, whereas the IgG-rich samples were sometimes associated with antibody-dependent enhancement (ADE) of replication. Accordingly, we could discriminate between ADE-positive and ADE-negative PRRSV strains. Next, we separated Ig isotypes in OF samples of PRRSV-infected pigs by means of protein A and size exclusion chromatography. The above results were confirmed by using separated Ig isotypes. Both dimeric and monomeric IgA were associated with the strongest reduction of PRRSV replication. The treatment of pig macrophages with separated OF antibodies before PRRSV infection was also associated with PRRSV yield reduction, along with clear changes of both CD163 and CD169 surface expression. Our results point at a role of mucosal IgA in the control of PRRSV replication by extra- and/or intracellular interaction with PRRSV, as well as by induction of signals leading to a reduced susceptibility of macrophages to PRRSV infection.

## Introduction

Porcine Reproductive and Respiratory Syndrome (PRRS) affects farmed pigs worldwide. It is sustained by two enveloped, positive-strand RNA viruses of the Arteriviridae family, genus Porarterivirus, including PRRSV-1, PRRSV-2 (30–45% variation in nucleotide sequences), Lactate dehydrogenase-elevating virus and Rat Arterivus 1 [1]. The two swine Arteriviruses had been previously identified as European (EU) type I, with the first strain isolated in 1991 and named “Lelystad”, and the North American (NA) type II, isolated in 1992 with the acronym ATCC VR-2332 [2]. Several disease signs can be detected on farm depending on pig age and production phase [3]. Although eradication might be feasible on the basis of herd closure with strict disease control and biosafety measures [4], the control of PRRS is usually based upon farm management procedures aimed at „stability“, i.e. a condition in which clinical signs of PRRS are absent in the breeding-herd population, and PRRSV is no more transmitted from sows to their offspring [5]. The absence of PRRSV in suckling piglets is of paramount importance, having in mind the much higher susceptibility of non-adult pigs to PRRSV and the much longer persistence of PRRSV in convalescent, non-adult pigs [6]. The foundation of a PRRS-stable farm is a successful “acclimatization” of replacement gilts and sows towards the PRRSV strains circulating in the farm before the breeding period. Pending the definition of reliable correlates of protection, “acclimatization” should be interpreted as a stepwise process of “adaptation” to field PRRSV strains, in which undefined immunological responses, down-regulation of permissiveness to PRRSV replication of pig macrophages and, perhaps, “education” of macrophages to a better control of inflammatory responses concur to obtain a pig population experiencing PRRSV infection without serious clinical outcomes.

The ontogeny of PRRSV-specific antibody in serum and oral fluids has been described using isotype-specific ELISAs [7]. Also, the mucosal IgA response of gilts is associated with a dramatic block of PRRSV spread in oral fluids (OF), whereas peak IgG responses are associated with virus shedding [8]. These peculiarities of PRRSV-specific IgA and IgG were confirmed in PRRS-stable farms with widely different acclimatization protocols; they were absent instead in two PRRS-unstable farms, which showed, poor and delayed (if any) IgA responses in OF [8].

This led us to postulate a role of mucosal IgA in the control of PRRS virus infection. In particular, our working hypothesis implied a role of mucosal IgA antibody in preventing PRRSV replication, and/or in modulating macrophage susceptibility to PRRSV. These features were investigated *in vitro* using pig macrophage cells, selected PRRSV strains isolated from clinical cases and OF samples with known PRRSV-specific, IgA and IgG antibody titers.

## Materials and methods

### Study design

Our study was organized in the following phases:

- Screening of OF samples from a large farm for IgA and IgG antibody to PRRSV.
- PRRSV yield reduction assays with OF samples at high, medium and low IgA content.
- Separation of Ig isotypes from PRRSV antibody-positive OF fluids and new yield reduction assays.
- Treatment of pig macrophages with separated Ig isotypes from OF samples and evaluation of their susceptibility to PRRSV replication.
- Assessment of IgA internalization in pig macrophages.

### Samplings

The study was carried out on nine groups of weaner and fattener pigs of ten pigs each of the same farm, and on 4 groups of pigs (total: 18) of a PRRS vaccine trial. All of these animals provided group OF samples. Eight SPF, PRRS-free pigs provided peripheral blood mononuclear cells (PBMC) for macrophage culture and differentiation, as well as for IgA internalization assays. A further SPF, PRRS-free, 2-month old pig was used to obtain Pulmonary Alveolar Macrophages (PAM) by broncho-alveolar lavage.

This study complied with Italian laws on animal experimentation and ethics. In particular, no animal experiment was carried out. All the PBMC and PAM samples had been obtained in previous studies and stored in liquid nitrogen. OF samples were obtained from our diagnostic department among specimens sent for investigating IgA and IgG antibodies to PRRSV (IZSLER test methods 04/117 and 04/118), and also from a previous safety study of a PRRSV vaccine strain.

### Cells and PRRSV strains

PBMC had been obtained by diluting heparinized blood samples 1:2 in RPMI 1640 medium and centrifuging (1100 *g*, 25’, 20°C) 9 mL of diluted blood on 3 mL of Histopaque ^®^-1077 (code 10771, Sigma-Aldrich). Thawed PBMC were washed once (330 g, 10’, 20°C) in RPMI 1640 medium and grown at 6x10^6^ / mL in RPMI 1640 medium at 37°C in 5% CO_2_, in microtiter plates over 2.5 hours. Next, non-adherent cells were discarded and monocytes were further cultivated under the same conditions in RPMI 1640 medium supplemented with 10% Fetal Calf Serum (FCS) and 10 ng/mL human Macrophage Colony Stimulating Factor (hM-CSF) (Macrophage Colony-Stimulating Factor human, code M6518-10UG, Sigma-Aldrich) over 3 to 5 days.

Pulmonary Alveolar Macrophages (PAM) had been collected in a previous study by broncho-alveolar lavage of a healthy, PRRS-naïve pig, as previously described [9]. As such, these cells were deemed representative of PRRSV-susceptible animals without the disturbing effects of environmental microbial pathogens. PAM were thawed on the day before the experiment and grown in microtiter plates at 10^6^/mL in RPMI 1640 medium supplemented with 20% Fetal Calf Serum (FCS) at 37°C in 5% CO_2_. These cells are not adherent to plastic and can be easily recovered by gently pipetting. One PAM batch only was used in all the experiments to minimize the variability of test results. More than 90% viability was always detected after thawing the frozen vials.

Cell cultures were checked for bacterial contaminations in Tryptic Soy and Thioglycolate broth media. Mycoplasma contaminations were investigated by fluorescent Hoechst 33258 staining, as previously described [10] (IZSLER internal method 01/088).

PRRSV strains were obtained from field cases of either respiratory or reproductive disease (Table 1). The presence of PRRSV was confirmed by Real time RT-PCR as described in the following section. ORF 5 and 7 genes of these strains were sequenced, and the relevant gene bank accession numbers are reported in Table 1.

**Table 1:** PRRSV strains included in the study.

PRRSV strains	Clinical cases on farm	Specimens	PCR results on specimen
270433–5	Abortion	blood	ND
(MN617072; MN617066)			
271009–8	Respiratory form	blood	ND
(MN617074; MN617068)			
3400/2	Abortion	blood	Ct 16
(MN617075; MN617069)			
13957	Abortion and respiratory form	lung homogenate	Ct 20
(MN617076; MN617070)			
21377	Abortion and respiratory form	lung homogenate	Ct 22.7
(MN617077; MN617071)			

The above strains are sometimes reported in a shortened form for convenience.

ND: not done. Ct: cycle threshold. Gene bank accession numbers for ORF 5 and ORF 7 genes (left and right, respectively) are reported between brackets.

### Real time RT-PCR for PRRSV ORF 7

A total of 200 μL of sample (serum or lung suspension) was subjected to RNA extraction using a commercial kit (NucleoMag® Vet kit, Macherey-Nagel, Düren, Germany), following the manufacturer’s instructions. To control the presence of inhibitors and success of the extraction, an exogenous internal control RNA (Xeno^TM^ RNA control, Applied Biosystem), was included with the specimens. The extraction was carried out on the Biosprint 96 instrument (Qiagen, Hilden, Germany), using the NucleoMag Vet 200 protocol. Nucleic acids elution was performed in 100 μL of elution buffer and the LSI VetMAX ^**(TM)**^ PRRSV EU/NA Real-Time PCR kit (Applied Biosystem), was used to detect the viral RNA.

Ten-fold serial dilutions in nuclease-free water of a EU PRRSV RNA (3.5 × 10^5^ to 35 copies/μL) were used to generate a standard curve and to quantify PRRSV RNA in the samples. Triplicates of each dilution were run in each assay. The following equation: $x=10\frac{cq-(yintercept)}{slope},$

where *x* represents the genome copies/μL, was used to transform the samples’ Cq values into estimates of genome copies of PRRSV RNA per mL of sample.

The detection limit of PRRSV-1 target was 9 copies of nucleic acids per reaction, corresponding to 0.642 genome copies/μL.

### Propagation of PRRSV strains

PRRSV strains from field cases (Table 1) were diluted 1:10 in RPMI 1640 medium + 2% FCS and filtered through 0.2 micron membranes. Next, 0.2 mL of each virus strain was adsorbed onto 3 to 5-day old adherent macrophages from PBMC in 6-well microtiter plates. After 2 hours at 37°C, 5% CO_2_, the inoculum was discarded and cells were washed twice with RPMI 1640 medium. Next, 4.5 mL of RPMI 1640 medium + 2% FCS were added to each well and plates were again incubated at 37°C, 5% CO_2_ for 1 hour. Then, 0.5-mL supernatant samples were collected from each well and stored at -80°C (day 0 samples). Plates were again incubated at 37°C, 5% CO_2_ and visually examined for cytopathic effects (cpe) over 2–3 days. Next, plates were set at -80°C for 2 hours at least before thawing and collecting again 0.5-mL samples from each well. Amplification of PRRSV strains was evaluated on the basis of cpe and quantified by Real time RT-PCR.

The same procedure was also applied to 1-day old, non-adherent PAM cultures in microtiter plates. In this case, cells were washed by centrifugation (330 g, 5 minutes, 20°C) after virus absorption.

### Isotype-specific indirect ELISA for antibody to PRRSV

OF samples were obtained from nine groups of pigs of different age (10 animals / group), housed in different pens of the same farm. OF were collected by a cotton thread cord with a frayed end hanging at about 5 cm from the snout of the animals. The cord was then placed into a stomacher bag, and transported to the laboratory under refrigerated conditions. It was manually squeezed and the collected liquid was centrifuged at 1,800 rpm for 10 minutes at 5°C; the supernatant was recovered into test tubes and frozen at -20°C until the analysis.

The concentration of PRRSV-specific antibody in oral fluids was measured by ELISA using a commercial kit (Herdcheck PRRS X3 Antibody Test Kit, IDEXX), as previously described [8]. Briefly, the kit was adapted to oral fluids by introducing HRP-conjugated, anti-swine IgG and IgA antisera, respectively, in the test procedure (Bethyl Laboratories Inc., Montgomery, Texas, catalog A100-104 and A100-102), and properly diluting the positive and negative controls of the kits to reach the usual threshold OD values. Also, as opposed to serum samples, 1:2-diluted oral fluids were reacted overnight at 4°C with PRRSV Ag-coated strips. This procedure had been validated on oral fluids of pigs collected before and after field infection with different PRRSV strain and compared with serum antibody titers [8]. On the basis of our experimental data, a sample to positive (s/p) ratio of 0.4 was identified as threshold for IgG and IgA-positive OF samples. s/p values < 0.4 were scored negative. The IgG and IgA-specific s/p values of each sample were compared. The Ab response was considered unbalanced if the IgA-specific s/p value was ≤ 50% of the IgG-specific one. This ELISA measures antibody to PRRSV N protein, which is a major target of the pig’s antibody response to PRRSV, for which major antigenic determinants are relatively well conserved amongst strains from the same continent [11]. As such, this kind of response was considered as a reliable index of the global IgA and IgG antibody responses in OF fluids. The neutralizing activity of OF samples against the PRRSV strains under study could not be checked, because these could not be adapted to MARC-145 cells, used in our neutralization assay [12].

### Separation of immunoglobulin (Ig) isotypes in pig OF samples

OF samples were classified in terms of (i) s/p values for IgA and IgG antibody, and (ii) IgA/IgG s/p ratio by our ELISA procedure. Samples with known IgA/IgG ratio were clarified by centrifugation (10,000 rpm, 10 minutes, 4°C) and filtered through 0.45 micron membranes. Dimeric IgA and total monomeric Ig were separated by means of gel filtration chromatography. Samples were run on a Superdex 200 10/300 GL column (GE Healthcare) under HPLC conditions in an AKTA Purifier apparatus (GE Healthcare). The column was calibrated with Relative Molecular Mass (Mr) standards ranging from Ribonuclease A (Mr 13,700) to Thyroglobulin (Mr 669,000). Samples (0.5 mL) were run at 0.75 mL/minute at room temperature in 0.01 M phosphate buffer, 0.14 M NaCl, pH 7.4. The void volume corresponded to 8 mL. Twenty-six, 0.5-mL fractions were collected and tested by our ELISA for IgA and IgG antibody. This enabled us to detect the fractions corresponding to dimeric IgA and monomeric IgG+IgA, respectively.

The same samples were also submitted to Protein A affinity chromatography. This enabled us to separate IgG antibody (eluted fraction) from non-IgG antibody in the unbound fraction (total IgA and IgM). The separated Ig fractions obtained by chromatography are summarized in Table 2.

**Table 2:** Immunoglobulins separated in oral fluid samples of pigs.

Procedure	Fractions	
Gel filtration chromatograghy	1) Dimeric IgA	2) Monomeric IgG / IgA
Protein A affinity chromatography	1) IgG antibody (eluted Ig)	2) Non-IgG antibody (IgA, IgM: unbound fraction)

### Yield reduction of PRRSV in pig macrophages

OF samples with known s/p values of anti-PRRSV IgA and IgG antibody were employed in a yield reduction assay of PRRSV in pig macrophage cultures grown in 48-well microtiter plates. Macrophages were obtained from pig monocytes as described in a previous section. Pre-titered, total OF samples with known s/p values of PRRSV-specific IgA and IgG antibody or Ig separated fractions (see previous section) were diluted 1:4 with PBS supplemented with Penicillin (250 micrograms/ml), Streptomycin (250 micrograms/ml), Amphotericin B (10 micrograms/ml) (ThermoFisher Scientific, catalog numbers 15140122 and 11805017), i.e. at five-fold increased concentrations with respect to tissue cultures in our lab. The final volume was 1.6 ml. Diluted OF samples were stored overnight at 4°C and centrifuged at 14,000 rpm in a Jouan BR4i centrifuge. Nanosep 10k omega centrifugal devices (Pall Corporation) were treated with 0.5 ml of 70% ethanol and centrifuged (14,000 rpm, 5 minutes). Ethanol was discarded and the procedure was repeated with 0.5 ml of sterile distilled water. Then, clarified OF samples were loaded on Nanosep devices and centrifuged at 10,000 rpm to restore the original 0.4 ml volume. The sample was diluted 1:4 with RPMI 1640 medium + 2% FCS + antibiotics and again brought back to the original 0.4 ml volume by centrifugation.

Each PRRSV strain was diluted in RPMI 1640 medium + 2% FCS + antibiotics to a concentration of 5x10^6^ genome copies/ml. Next, 125 microliters of PRRSV were mixed with 125 microliters of 1:2-diluted OF sample. After 1 hour at 37°C, 100 microliters/well of the OF/PRRSV mixture were adsorbed onto pig macrophages and incubated at 37°C, 5% CO_2_ for two hours. The inoculum was discarded, cells were washed twice with RPMI 1640 + 2% SFB and 0.75 ml /well of the same medium was added. After a further 60 minutes at 37°C, 5% CO_2_, a volume of 0.25 ml/well was harvested and frozen at -80°C as sample A, representing the background of non-adsorbed PRRSV. The plates were incubated for two more days at 37°C, 5% CO_2_, and cpe was monitored at 24-hour intervals. Finally, a second sample B was collected to verify the replication of PRRSV in the macrophage culture. This was verified by Real time RT-PCR as described in a previous section. Then, the difference (Delta) between A and B values (genome copies and Ct value) was reckoned. Yield reduction of PRRSV was evaluated according to either formula:

- [genome copies/ml in control cells (B-A)–genome copies in OF-treated cells (B-A)].
- [DeltaCt (A-B) in control cells)–DeltaCt (A-B) in OF-treated cells)]

The above procedure was also applied to PAM cultures in 48-well tissue culture plates. In this case, PAM cultured in 48-well microtiter plates were centrifuged at 330 g, 5 minutes, 20°C. The supernatant (350 microliters) was removed and the virus/OF mixture was added. After 2 hours at 37°C, the plates with cells were washed twice (330 g, 5 minutes) and supplemented with 700 microliters/well of RPMI1640 medium + 10% FCS. After a further 60 minutes at 37°C, 0.25 ml/well (A sample) were collected and stored at -80°C. The plates were incubated for two more days at 37°C, 5% CO_2_, and cpe was monitored at 24-hour intervals before collecting the B samples. In case of separated Ig fractions, these were read spectrophotometrically at 280 nm to assess protein concentration and fractions were diluted in tissue culture medium to obtain the same total OF protein concentration.

### Modulation of macrophage permissiveness to PRRSV infection

In order to evaluate Ig-driven modulation of pig macrophage susceptibility to PRRSV, PAM and macrophages from blood monocytes in 48-well microtiter plates were treated with 50 microliter/well of separated Ig fractions for 1 hour at 37°C. Cells were washed twice with RPMI + 10% FCS and then infected with PRRSV under the same conditions of the yield reduction assay.

### Flow cytometry analyses

Plastic adherent macrophages from blood monocytes were washed twice with Ca and Mg-free PBS, and detached in EDTA 10 mM in PBS over 1 hour at 4°C by gently scraping with the tip of a micropipette. After washing twice in flow cytometry buffer (FCB, i.e. PBS + 2% heat-inactivated FCS + 0.1% azide), cells were resuspended at 6x10^6^ / ml in FCB. Fifty microliters/well of this suspension were distributed in 4 wells of a 96-well, U-bottomed microtiter wells. Cells of two wells were reacted with 25 microliters of pre-titered monoclonal antibodies 2A10/11 to porcine CD163 (BIO-RAD), 3B11/11 to porcine CD169 (BIO-RAD) respectively, involved in binding and internalization of PRRSV [13]. A few samples were also stained with Mouse anti-Pig SLA Class II DR: FITC (BIO-RAD, clone 2E9/13, cat. MCA2314F) to porcine MHC II. Two wells were reacted with FCB, only (control). After 30 minutes at 4°C, 100 microliters/well of FCB were added and plates were centrifuged at 500 g, 3 minutes. The plates were flicked over a sink and all wells but one (cell control) were reacted with 25 microliters /well of Alexa Fluor® 488 F(ab')2 fragment of goat anti-mouse IgG, IgM (H+L) (Thermo Fisher, cat. A10684). After 30’ at 4°C, cells were washed twice (500 g, 3’) with 100 microliters/well and resuspended in 200 microliters/well FCB. 2 microliters/well of Propidium iodide (PI) at 50 micrograms/ml (BD Pharmingen cat. 51-66211E) were added and 10,000 events were analyzed in a Guava EasyCyte HT flow cytometer using Incyte software. The gating strategy is shown in Fig 6. Green fluorescence of PI-negative (viable) cells was evaluated with respect to the secondary antibody control, whereas forward and side scatter signals were calibrated on the basis of the unreacted control cells.

Intracellular IgA was investigated on peripheral blood monocytes from three PRRS-free, SPF sows (named 1, 2, 3), differentiated to macrophages in 24-well microtiter plates as described above over 4 days. On the 3^rd^ day, the non-IgG fraction of OF (see Table 2) was supplemented overnight at 4°C with an antibiotic mixture (Penicillin at 250 micrograms/ml, Streptomycin at 250 micrograms/ml, Amphotericin B at 10 micrograms/ml, final). After centrifugation (14000 rpm, 15’, 4°C), 120 microliters of the OF fraction was mixed with the same volume of PRRSV strains 957 and 009/8 (5x10^6^ genome copies /ml), and medium (RPMI 1640 + 10% SFB), respectively. After 1 hour at 37°C, the virus/OF, medium/OF and medium only (control) samples were transferred onto adherent macrophages. After 1 hour at 37°C in 5% CO_2_, the samples were discarded, macrophages were washed twice with PBS and detached in PBS-10mM EDTA (1 hour at 4°C), fixed in 3% formaldehyde, permeabilized in PBS-1% saponin (PBS-S), and submitted to blocking of Fc receptor sites by incubation with PBS-S supplemented with 10% goat serum (20 minutes at room temperature). Next, they were reacted (20 minutes at room temperature) with a mAb to swine IgA (AbD Serotec, cat. MCA638), washed and further reacted with Alexa Fluor® 488 F(ab')2 fragment of goat anti-mouse IgG, IgM (H+L) (Thermo Fisher, cat. A10684) in PBS-S. After two further washings in PBS-S and a last one with PBS, respectively, cells were resuspended in FCB for flow cytometry analysis of intracellular IgA. The gating strategy is shown in Supplementary file 2.

### ELISA for intracellular IgA

Pig macrophages from blood monocytes were treated with the non-IgG fraction of OF (see Table 2) or medium only (control), as described for the IgA flow cytometry assay (see previous section). Next, cells were detached with EDTA 10 mM, pelleted, resuspended in 200 microliters of TRIS buffered saline with 1% BSA, pH 8.0 (Sigma, code T-6789) + Protease Inhibitor Cocktail set III 1:200 (Calbiochem, code 539134), and submitted to two cycles of freezing and thawing at -80°C, followed by centrifugation at 10000 rpm, 10’, 4°C. The supernatants were tested for IgA according to an established procedure (Pig IgA ELISA Quantitation Set, Bethyl Laboratories, code E100-102), according to the manufacturer’s directions. A standard curve was created using a pig reference serum with a known concentration of IgA (Bethyl Laboratories, code RS10-107-4).

### Statistical analyses

Replication of PRRSV in terms of genome copies/ml was investigated by two-way ANOVA in tests on raw OF samples, including effects of both PRRSV strain and prevalent Ig type (IgA or IgG) in OF. Two-way ANOVA was also employed to investigate PRRSV replication in different macrophage populations (effects of macrophages and PRRSV strains). One-way ANOVA and Newman-Keuls Multiple Comparison test was adopted instead in yield reduction assays on separated Ig fractions. The prevalence of cells expressing CD163 and CD169 in flow cytometry analyses was evaluated by a Fisher’s exact test. The significance threshold was set P< 0.05. A tenden**cy** was declared at P< 0.1 (Graph Pad Prism 5, GraphPad Software Inc., La Jolla, CA).

## Results

### Cells

As expected, differentiated, CD163+ macrophages were not detected in PBMC of healthy, PRRS Ab-negative, Specific Pathogen Free (SPF) pigs, stored in liquid nitrogen (data not shown). Pig monocytes were readily differentiated into PRRSV-susceptible macrophages under the aforementioned conditions. The resulting cells turned larger, firmly adherent to plastic, and were unambiguously defined as monocyte-derived macrophages due to their CD163+, CD169+, MHC II+ phenotype (S1 Fig). PAM showed the same surface phenotype and also proved competent for PRRSV replication. In terms of macrophage population types, the resulting cells can be described as “neutral”, i.e. differentiated neither into M1, nor into M2 types [14]. In fact, it was our understanding that uncommitted macrophage populations could more closely reflect the *in vivo* situation, and avoid any bias in the interaction with the PRRSV strains under study.

### Yield reduction of PRRSV is affected by the IgA to IgG ratio in OF samples

**Fig 1:**
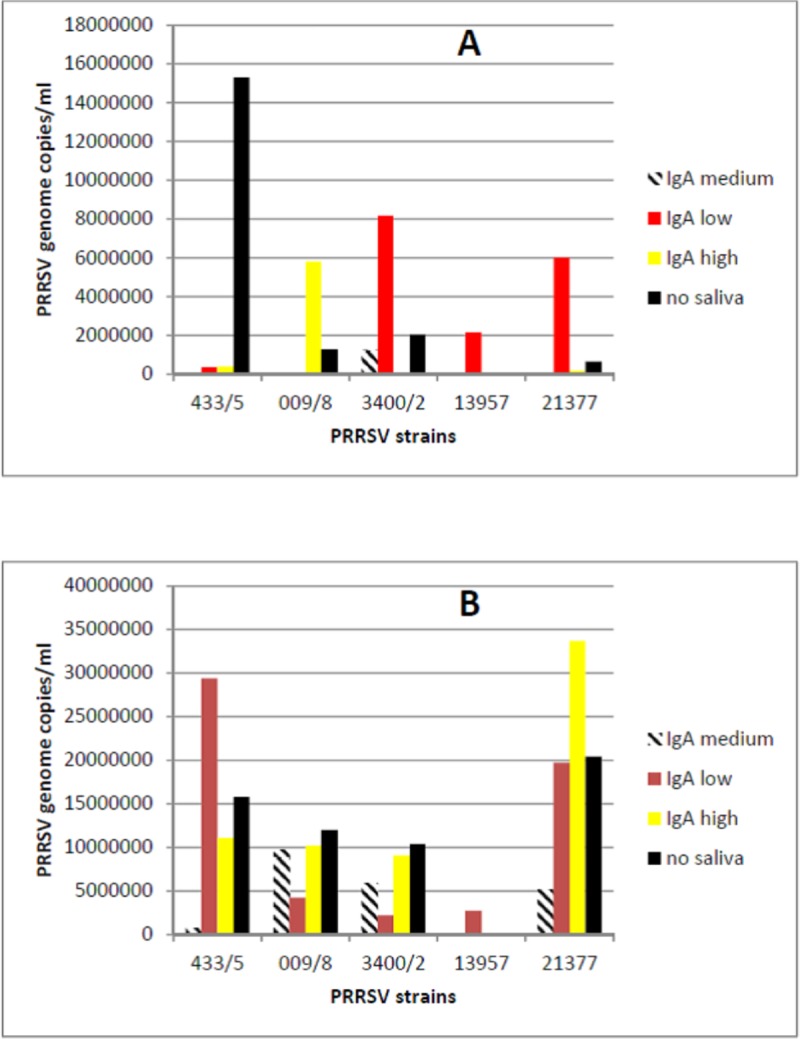
Yield reduction assays. Five PRRSV strains were reacted with IgA-low, IgA-medium, IgA-high OF samples, and with medium only (no OF), respectively. The replication of PRRSV in macrophages from blood monocytes was measured by Real time RT-PCR for PRRSV ORF 7 and expressed in terms of PRRSV genome copies / ml. Panel A: experiment 1. Panel B: experiment 2 on macrophages from another pig. P< 0.05 for PRRSV strain in experiment 2.

**Fig 2:**
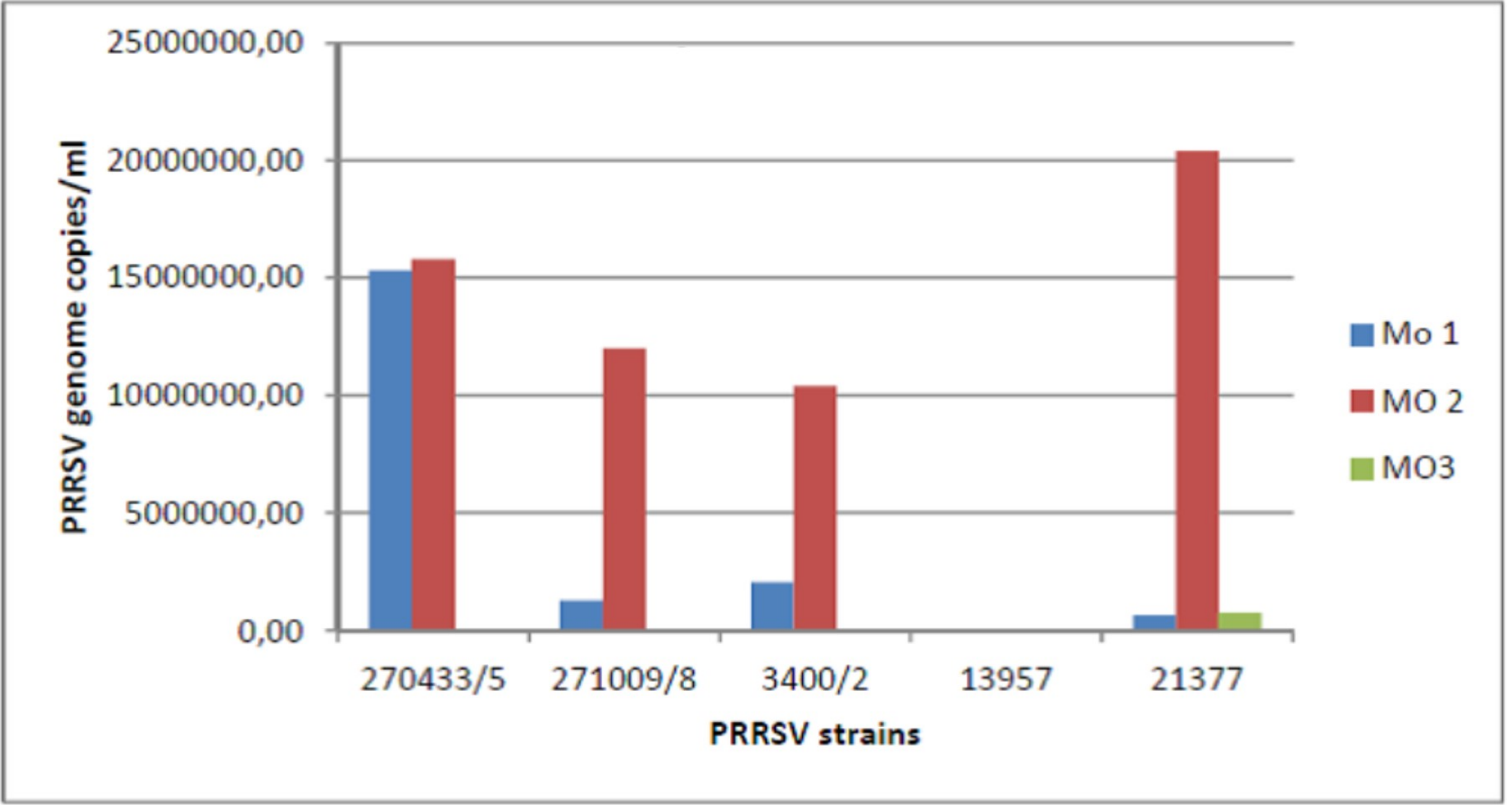
Permissiveness to PRRSV of pig macrophages. The replication of 5 PRRSV strains was investigated on macrophages from blood monocytes of three different pigs under the same culture conditions. The replication of PRRSV was measured by Real time RT-PCR for PRRSV ORF 7 and expressed in terms of PRRSV genome copies / ml. P< 0.05 for macrophage population.

We had previously shown that peak anti-PRRSV IgA titers correspond to a substantial block of PRRSV shedding in OF samples [8]. Therefore, we selected 9 OF samples showing widely different IgA to IgG ratios from the same PRRSV-positive farm, in which pigs had been exposed to the same PRRSV field strains. We eventually chose three of them, showing high (1.27), medium (0.91) and low (0.60) IgA/IgG s/p ratios, respectively. In the first experiment, these OF samples were employed in a yield reduction assay on 5 PRRSV strains using macrophages obtained from monocytes of the same pig (Fig 1, panel A). The PRRSV strains under study showed wide differences of replication in the control cultures. The largest yield reduction was obtained with the IgA-medium sample on 4 samples out of five, whereas the IgA-low sample caused antibody-mediated enhancement (ADE) of replication with 3 PRRSV strains. In particular, moderate replication of PRRSV strain 13957 was only shown in the presence of the IgA-low OF sample. In a second experiment on macrophages from another pig, there was less yield reduction on average, caused by IgA-medium and IgA-low OF on two strains each, whereas ADE was caused once again by the IgA-low OF for PRRSV strain 13957 (Fig 1, panel B; P< 0.05 for PRRSV strain). PRRSV strain 009/8 differed from the other ones since it was inhibited by IgA-low OF and caused ADE with IgA-high OF (Fig 1 A). Most important, there was evidence that PRRSV strains and also pig macrophage populations played a major role in the results of the yield reduction assay. To confirm this tenet, we investigated PRRSV replication in a third experiment on macrophages from monocytes of three other pigs using the same PRRSV strains. Results are shown in Fig 2. Despite the same culture conditions, we observed clear differences of replication among the five PRRSV strains and the three pig macrophage populations (P< 0.05 for macrophage population). On the whole, our results were based on three experiments carried out on macrophages from five different pigs.

### Separation of pig Ig isotypes in OF samples

**Fig 3:**
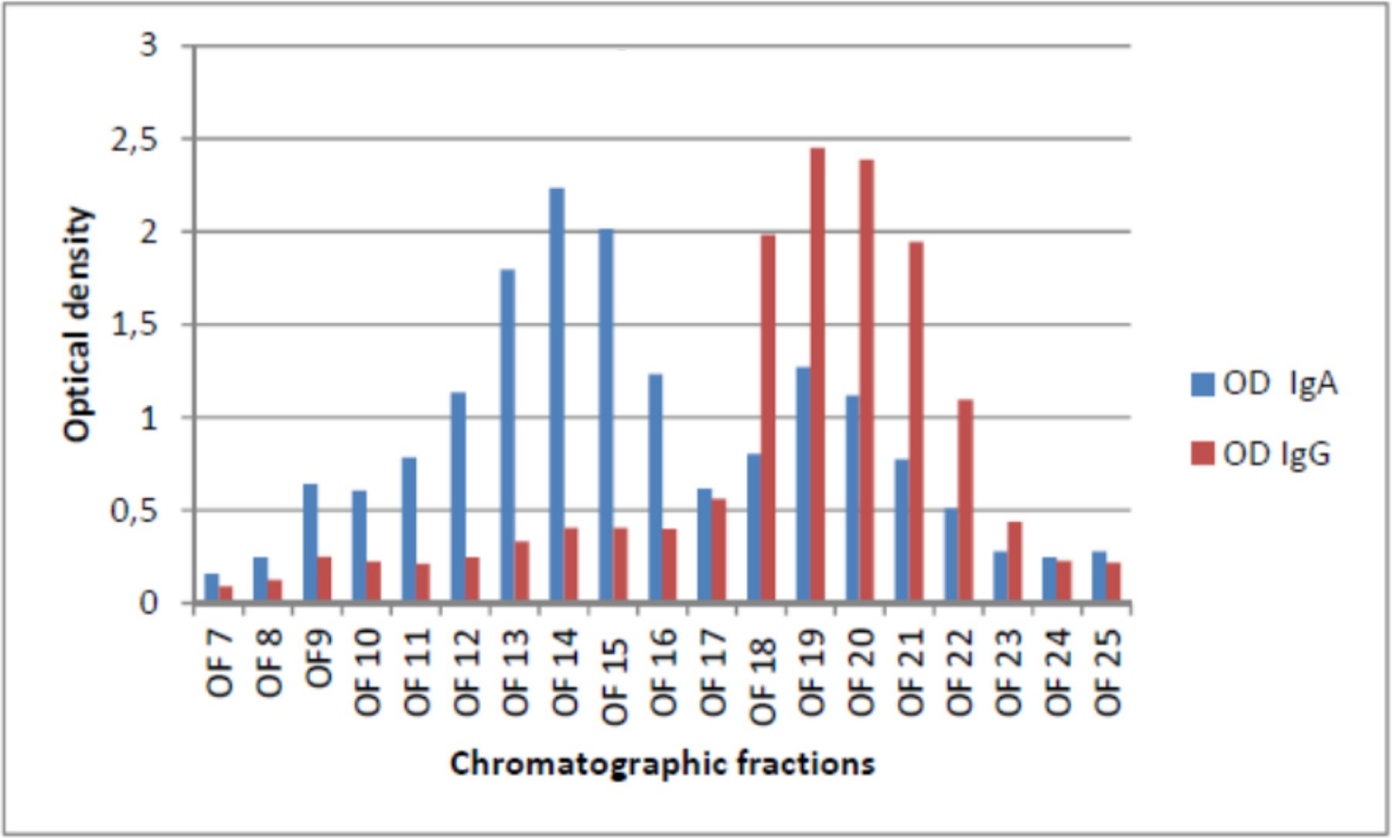
Separation of Ig fractions in OF samples. Dimeric IgA and total monomeric IgA + IgG were separated by means of gel filtration chromatography. Samples were run on a Superdex 200 10/300 GL column (GE Healthcare) under HPLC conditions in an AKTA Purifier apparatus (GE Healthcare). Twenty-six, 0.5-mL fractions were collected and tested by ELISA for IgA and IgG antibody, respectively. This enabled us to detect the fractions corresponding to dimeric IgA (12 to 16) and monomeric IgG+IgA (18 to 21), respectively.

Mucosal dimeric IgA could be separated from monomeric IgG and IgA by gel filtration chromatography on pools of OF samples, whereas IgM antibody (around 900 kDa) was included in the void volume. Our IgA and IgG-specific ELISA test for antibody to PRRSV enabled us to detect the fractions of interest (see Fig 3 and Table 2). These were employed in yield reduction assays along with the two fractions of Protein A affinity chromatography (IgG and non-IgG antibody, Table 2). On the basis of this procedure, five tests were carried out on separated Ig fractions from OF samples.

### IgA-positive OF fractions cause yield reduction of PRRSV in pig macrophages

**Fig 4:**
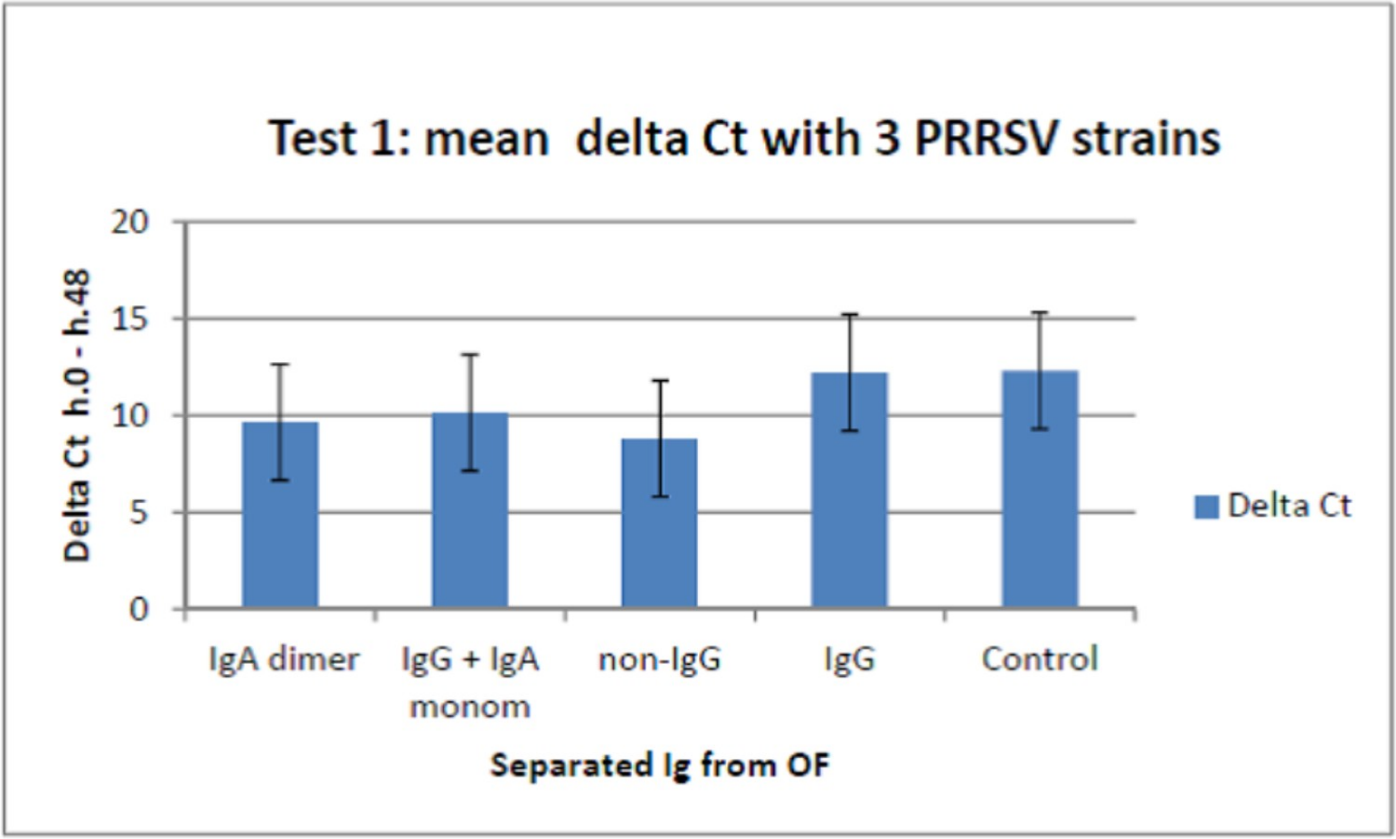
Yield reduction assay in PAM, test 1. Ig fractions were separated from a pool of antibody-positive OF samples (s/p IgA: 2.73; s/p IgG: 3.13) and used in a yield reduction assay on PAM. Results are shown in terms of mean genome copies/ml for PRRSV strains 433/5, 009/8 and 377 ± 1 standard deviation. Separated Ig significantly differed for antiviral activity (P< 0.001; P< 0.05 for non-IgG fraction vs. IgG, and monomeric IgA/IgG vs. IgG).

In test 1, we separated Ig from a pool of the nine antibody-positive OF samples used in the first screening, as specified above (s/p IgA: 2.73; s/p IgG: 3.13). Separated Ig showed different levels of yield reduction of PRRSV strains 433/5, 009/8, 377 in PAM, the highest activity being provided by IgA-containing fractions (P< 0.001, Fig 4). Interestingly, these results were not confirmed on PRRSV strain 957, on which both IgA and IgG fractions caused little if any yield reduction.

**Fig 5:**
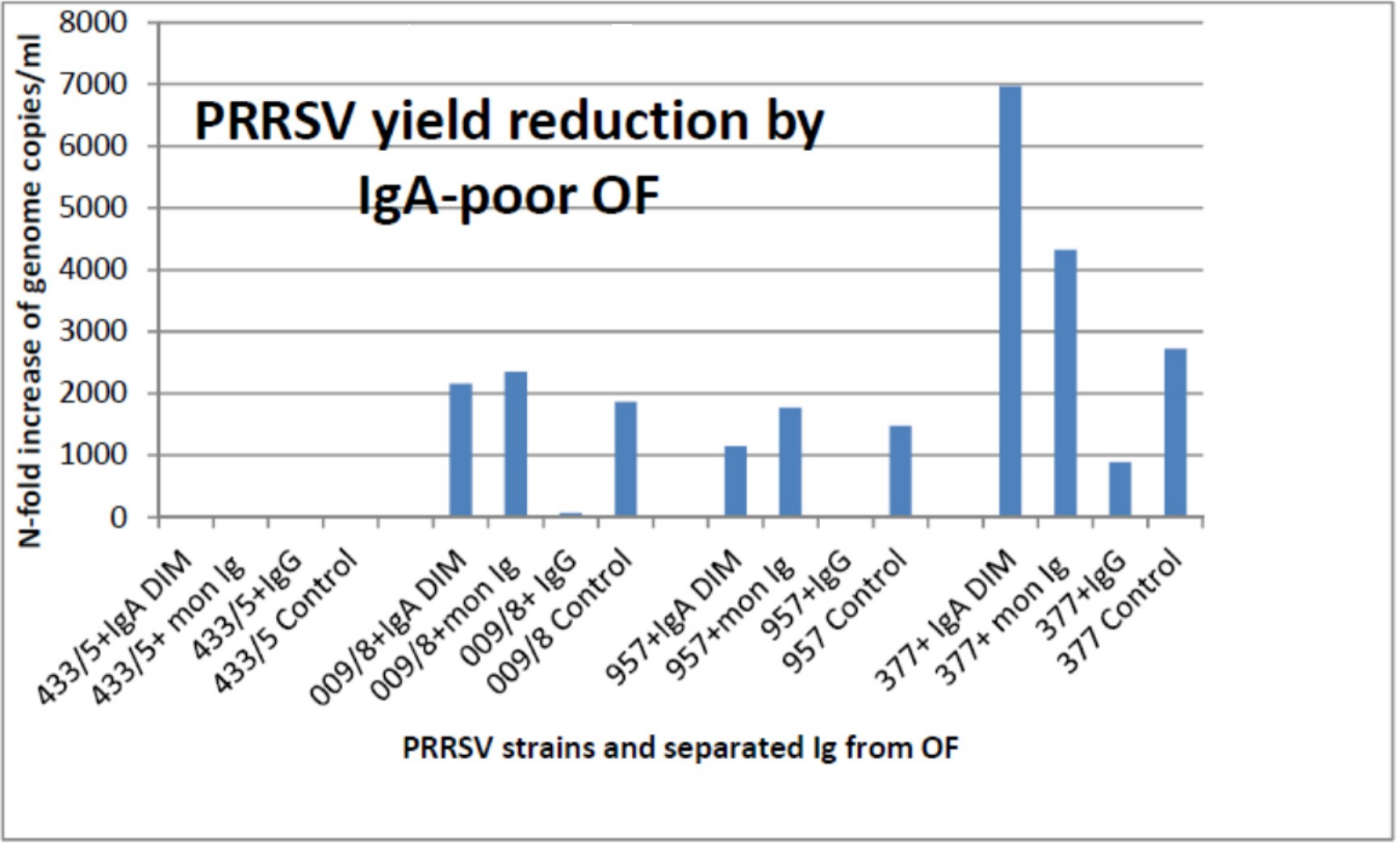
Yield reduction assay in PAM, test 2. Ig fractions were separated from an OF pool showing little if any IgA response (s/p IgA: 0.63; s/p IgG: 1.54). These IgA-low fractions exerted by far less or no antiviral activity on strains 009/8, 377 and 957, compared with the IgG fraction, whereas strain 433/5 replicated very poorly (P< 0.1, tendency).

In test 2, we deliberately prepared the same Ig fractions from another OF pool showing little if any IgA response (s/p IgA: 0.63; s/p IgG: 1.54). As opposed to the first OF pool, the IgA-containing fractions exerted by far less or no antiviral activity on strains 009/8, 377 and 957, compared with the IgG fraction, whereas strain 433/5 replicated very poorly (Fig 5). This led us to think that IgA concentration and affinity in OF affected the final results of our yield reduction assay.

Test 3 was a repetition of test 1. A confirmation of the previous results was obtained for the non-IgG fraction (strongest inhibition of strains 009/8, 377) but not to the same extent observed in Test 1 (P for Ig type > 0.1).

**Fig 6:**
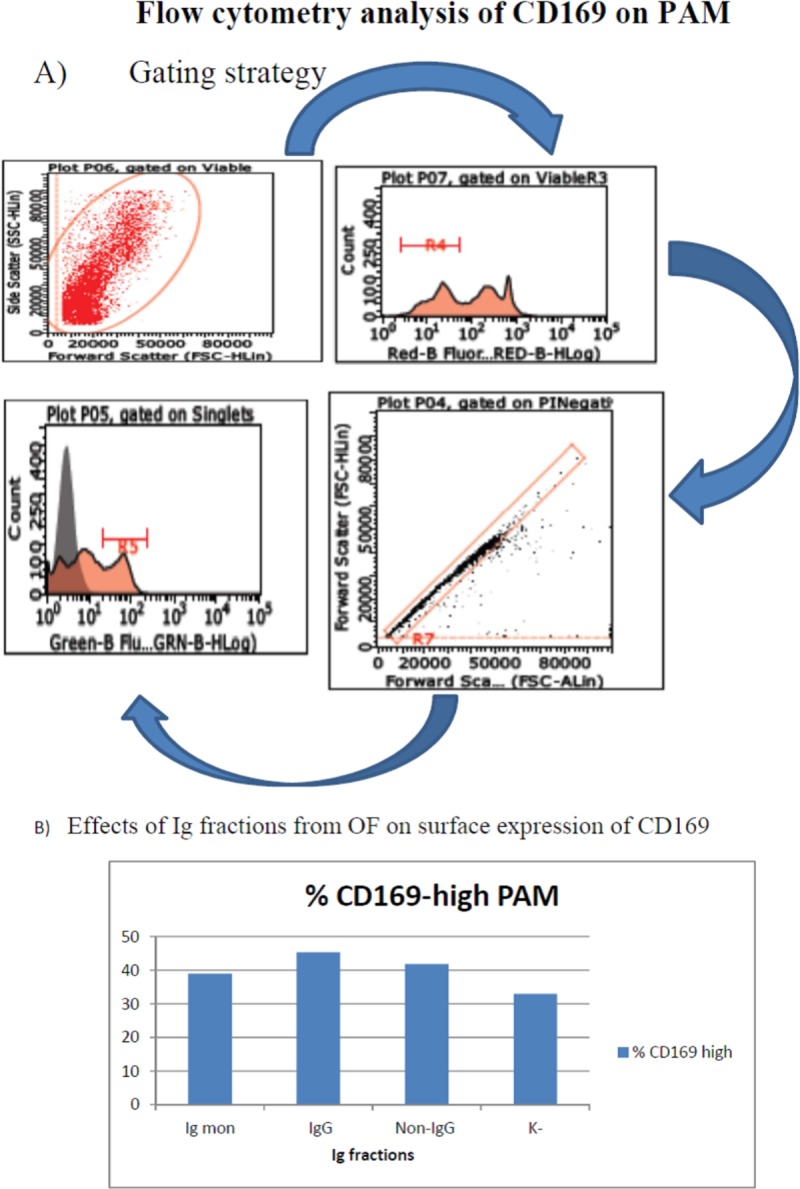
Surface expression of CD169 in PAM, test 4. The direct interaction of IgG, monomeric IgA+IgG and non-IgG fractions with PAM was investigated. Panel A: gating strategy. PAM were gated first by a combination of forward and side scatter. Next, viable cells were selected by staining with PI (red fluorescence), and submitted to singlet analysis. CD169+ PAM were detected by monoclonal antibody 3B11/11 and Alexa Fluor® 488 F(ab')2 fragment of goat anti-mouse IgG, IgM (H+L). Panel B: prevalence of CD169-high PAM is depicted in a bar graph. The three Ig fractions caused up-regulation of CD169 in PAM (P< 0.001 with respect to control cells).

In test 4, we evaluated the direct interaction of IgG, monomeric IgA+IgG and non-IgG fractions with PAM, using the same OF pool used in Tests 1 and 3. The three Ig fractions caused a significant up-regulation of CD169 in PAM (Fig 6); yet, only IgG and mon Ig caused up-regulation of CD163, as opposed to the non-IgG fraction.

The above findings (Test 4) had shown a direct role of OF antibody in the modulation of surface receptors involved in PRRSV replication. We wondered if this could affect PRRSV replication without a direct interaction between antibodies and PRRSV. Therefore, we prepared two other pools of OF samples, deriving from control and PRRSV-infected pigs, respectively. Antibody-positive OF samples had been collected 12 and 20 days after an experimental PRRSV infection and showed s/p titers > 2 for both IgA and IgG antibody to PRRSV. This was an early Ab response to PRRSV, as opposed to the Ig fractions used in tests 1 and 3. In Test 5, the replication of PRRSV was evaluated in pig macrophages from blood monocytes, treated with the above Ig fractions and then infected with PRRSV. The IgA-containing fractions of the OF Ab-positive pool caused PRRSV yield reduction, as opposed to the fractions from PRRS-free pigs. These caused ADE or no effect on PRRSV replication (Table 3). Interestingly, PRRSV strain 009/8 was inhibited by the IgG fraction of the Ab-positive pool, in agreement with the results shown in Figs 1 and 5. On the whole, PRRSV permissiveness could be modulated by Ig fractions in OF.

**Table 3:** PRRSV replication assay on macrophages derived from blood monocytes.

	PRRSV strain 433/5		Strain 009/8	
Ig fraction	OF+	OF-	OF+	OF-
IgA dim	13,800	316,000	624	2860
Ig mon	396,000	18,600	1300	49500
IgG	75500	4240	0	4,970,000
Non-IgG	8920	4,710,000	1430	110000

Test 5. Ig fractions from OF of PRRSV-infected and control pigs were reacted with macrophages derived from blood monocytes. Next, they were infected with PRRSV strains 433/5 and 009/8. Results are shown in terms of PRRSV genome copies/ml at 48 hours after infection.

### Internalization of IgA into macrophages

The above results (Table 3) led us to postulate that both free, PRRSV-specific IgA and IgA/PRRSV immunocomplexes could be internalized into PRRSV-susceptible pig macrophages. Using macrophages from SPF sow 3, IgA internalization as immuno-complexes was enhanced with PRRSV strain 957, compared with strain 009/8 and medium (S2 Fig). Instead, using macrophages from sows 1 and 2, no significant difference was demonstrated by flow cytometry. This was also confirmed by ELISA, even using a 10-fold higher concentration of PRRSV strain 957 (5x10^7^ genome copies/ml) (Table 4).

**Table 4:** ELISA for intracellular IgA.

Macrophages from sow:	Sample	ng/ml intracellular IgA
**1**	IgA + PRRSV 1X	**36**
	IgA + PRRSV 10X	**31**
	IgA + medium	**43**
**2**	IgA + PRRSV 1X	**39**
	IgA + PRRSV 10X	**38**
	IgA + medium	**35**

Macrophages from blood monocytes of sows 1 and 2 were reacted with the non-IgG fraction from test 4, either free or as immuno-complex with PRRSV strain 957 at two distinct concentrations. After washing, cells were detached, pelleted and frozen. Supernatants of frozen/thawed macrophages (2 cycles) were tested for IgA according to an established procedure (Pig IgA ELISA Quantitation Set, Bethyl Laboratories, code E100-102), according to the manufacturer’s directions. A standard curve was created using a pig reference serum with a known concentration of IgA (Bethyl Laboratories, code RS10-107-4). 1X: 5x10^6^ genome copies / ml; 10X: 5x10^7^ genome copies / ml.

## Discussion

PRRSV had circulated in farmed swine for decades without causing disease symptoms [15]. This favorable host/virus relationship changed dramatically in the eighties’ with the advent of lean type, rapid growth pigs; these are indeed more susceptible to PRRSV infection under experimental conditions [16]. Also, the global diffusion of PRRS among farmed pigs worldwide led to the selection of virulent virus strains, underlying serious disease epizootics and high economic losses [17].

Despite extensive research efforts, PRRSV infection remains an elusive model of host/virus relationship, in which no mechanism of adaptive immunity has been convincingly indicated as a reliable correlate of protection. Thus, neutralizing antibody may be effective for passive clinical protection before PRRSV infection [18], but it does not prevent PRRSV replication in target tissues nor transmission to susceptible animals [19]. On the contrary, no clear effect of antibody can be demonstrated in PRRSV-infected pigs. As an example, viremia may co-exist with neutralizing antibody for several weeks [20], and high-titered antibody responses can be even correlated with a worse clinical outcome of PRRS [21].

As for cell-mediated immunity, there is often a considerable delay of the PRRSV-specific IFN-gamma response [22], which is completely suppressed by PRRSV viremia [8]. In PRRS-naïve pigs, viremia may be very long, and its control is not correlated with any particular type of adaptive immune response [22]. The lack of effective, adaptive immune responses was also confirmed in the murine Arterivirus model, in which viremia has the same duration in virus-tolerant and immunocompetent mice [23]. On the whole, such findings led us to postulate that the host’s control of PRRSV infection would be simply the result of a stepwise lack of permissiveness of macrophages to PRRSV replication, as also suggested in a previous study [24]. This is the reason why we were somewhat surprised by the strong correlation between peak IgA response and cessation of PRRSV shedding in OF samples [8]. The issue of the mucosal IgA response to PRRSV is two-fold:

1. Is there an IgA-dependent inhibition of PRRSV replication in macrophages ?
2. Is such a mechanism due to interaction of IgA with PRRSV and/or with macrophages ?

The second query is accounted for by strong evidence liaising environmental stimuli on macrophages and susceptibility to PRRSV replication. In particular, inflammatory-type macrophages grown in the presence of LPS and IFN-gamma show reduced susceptibility to PRRSV, as opposed to macrophages grown in the presence of glucocorticoids and IL-10 [14]. In this respect, PAM may be sometimes representative of the former condition, because of the *in vivo* exposure to airborne LPS in swine farms [25], whereas blood monocytes are differentiated *in vitro* to macrophages in the absence of such stimuli. Interestingly, circumstantial evidence in our lab had shown poor sensitivity of PAM to PRRSV, if such cells were obtained from lungs with foci of pneumonia.

In this conceptual framework, our findings showed an IgA-driven inhibition of PRRSV replication in macrophages from blood monocytes. As a caveat, such a correlation was not as clear in PAM (compare results of Tests 1 and 3), which points at other, still undefined factors affecting PRRSV replication. In particular, this makes a case for an investigation into the expression of surface IgA receptors in both types of macrophages. Secondly, our yield reduction assays revealed the presence of ADE-positive and negative PRRSV strains under the same culture conditions. Thirdly, yield reduction by IgA was not strictly dependent upon a primary interaction with PRRSV, and presentation of immuno-complexes to pig macrophages from blood monocytes. Also, some PRRSV strains like 009/8 might be less susceptible to IgA-driven neutralization and be mainly inhibited by OF IgG. As opposed to strain 009/8, PRRSV strain 13957 showed ADE with IgA-low OF on macrophages from blood monocytes and the opposite on PAM (compare Figs 1 and 5). This outlines the crucial role of macrophage type and functional state, whereby Ab to PRRSV of the same isotype may be inhibiting or enhancing the replication of the same PRRSV strain. Finally, a note of caution should be expressed about the few OF pools available in our study: a more detailed knowledge will be gained in the future on the basis of larger samplings. In particular, the authors are aware that the limited results of this study cannot be extrapolated to final conclusions.

Pending the evaluation of further OF samples, our findings should be presently evaluated on the basis of the current knowledge about IgA receptors. In fact, monomeric and dimeric IgA can signal through different receptors: FcαRI (CD89), pIgR, Fcα/μ receptor, DC-SIGN, Transferrin receptor (CD71) [26]. As for macrophages, FcαRI is likely to play a major role: after contact with FcαRI, uncomplexed, monomeric IgA are conducive to ITAM-signaling (through associated FcRγ) and IL-10 responses, whereas macrophage activation and IFN-γ are triggered by IgA immuno-complexes and polymeric IgA [26]. IL-10 and IFN-γ responses underlie PRRSV-permissive and non-permissive MO phenotypes, respectively [14]. Instead, IgG immunocomplexes signal to Fcγ receptors and may cause ADE of PRRSV replication [27]. This is fully in agreement with our field study on Ab responses and PRRSV shedding in OF samples [8]. Also, the results of Test 4 indicate that a possible mechanism for ADE may be the up-regulation of CD163 induced by IgG antibodies, because of the crucial role of CD163 in PRRSV replication [13].

Mucosal IgA can also perform intracellular neutralization following interaction of cells with its Secretory Component (SC), as previously shown in the influenza model [28]. This mechanism can account for PRRSV yield reduction without a direct, extracellular virus/Ab reaction (see Tables 3 and 4). Therefore, the IgA response can cause direct extra and intracellular neutralization of PRRSV, and also reduce macrophage permissiveness to viral replication in the form of IgA/PRRSV immunocomplexes.

On the whole, the emerging picture in the PRRS model outlines unusual effector roles of adaptive immunity: both Ab and cell-mediated immune responses can concur to a major modulation of macrophage permissiveness to PRRSV. This also refers to the development of PRRSV-specific IFN gamma-secreting T cells [22], since IFN-gamma is likely to induce PRRSV-resistant pig macrophages [14]; the down-regulation of CD163 induced by IFN-gamma [13] is probably relevant to this latter effect.

## Conclusions

OF IgA can be involved in extra or intracellular interaction with PRRSV, and it can also convey signals leading to reduced permissiveness of macrophages to PRRSV infection. Therefore, a proper IgA response in oral fluids contributes to an effective control of PRRSV infection.

## Supporting information

S1 Fig Expression of CD163 in swine macrophages. Plastic adherent macrophages from blood monocytes were washed twice with Ca and Mg-free PBS and detached in EDTA 10 mM in PBS over 1 hour at 4°C after gently scraping with the tip of a micropipette. After washing twice in flow cytometry buffer (FCB, i.e. PBS + 2% heat-inactivated FCS + 0.1% azide), cells were resuspended at 6x10^6^ / ml in FCB and stained with monoclonal antibody 2A10/11 to porcine CD163 and Alexa Fluor® 488 F(ab')2 fragment of goat anti-mouse IgG, IgM (H+L). Orange-colored area: negative control. White-colored area: mAb 2A10/11-stained cells.
(PDF)

S2 Fig Intracellular IgA in swine macrophages. Peripheral blood monocytes from three PRRS-free, SPF sows were differentiated to macrophages over 4 days. Then the non-IgG fraction of OF from test 4 (see Results section) was reacted with PRRSV strains 957 and 009/8 (5x10^6^ genome copies /ml), and medium (RPMI 1640 + 10% SFB), respectively. After 1 hour at 37°C, the virus/OF, medium/OF and medium only (control) samples were transferred onto adherent macrophages. After 1 hour at 37°C in 5% CO_2_, the samples were discarded, macrophages were washed twice with PBS and detached in PBS-10 mM EDTA (1 hour at 4°C), fixed in 3% formaldehyde and permeabilized in PBS-1% saponin (PBS-S). Intracellular IgA were revealed with a mAb to swine IgA (AbD Serotec, cat. MCA638) and Alexa Fluor® 488 F(ab')2 fragment of goat anti-mouse IgG, IgM (H+L). **A:** macrophages gated by a combination of forward and side scatter. **B:** gating of singlets. **C:** staining of intracellular IgA in macrophages of sow 3.
(PDF)
