# Supplementary material for: Characterization of the IgA response to PRRS virus in pig oral fluids
Source: PLoS One. 2020 Mar 3;15(3):e0229065. doi: 10.1371/journal.pone.0229065 (PMC7053757; doi:10.1371/journal.pone.0229065)
Supplement: S1 Fig — Plastic adherent macrophages from blood monocytes were washed twice with Ca and Mg-free PBS and detached in EDTA 10 mM in PBS over 1 hour at 4°C after gently scraping with the tip of a micropipette. After washing twice in flow cytometry buffer (FCB, i.e. PBS + 2% heat-inactivated FCS + 0.1% azide), cells were resuspended at 6x10^6^ / ml in FCB and stained with monoclonal antibody 2A10/11 to porcine CD163 and Alexa Fluor® 488 F(ab')2 fragment of goat anti-mouse IgG, IgM (H+L). Orange-colored area: negative control. White-colored area: mAb 2A10/11-stained cells. (PDF) [file pone.0229065.s001.pdf]

S1 Figure

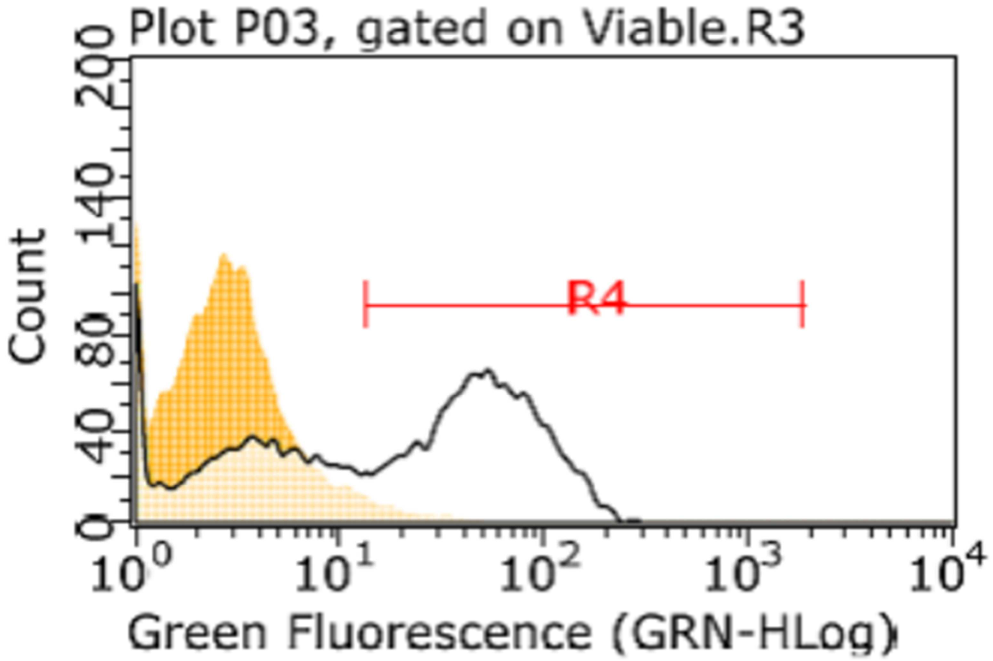

S 1 Figure. Expression of CD163 in swine macrophages. Plastic adherent macrophages from blood monocytes were washed twice with Ca and Mg-free PBS and detached in EDTA 10 mM in PBS over 1 hour at 4°C after gently scraping with the tip of a micropipette. After washing twice in flow cytometry buffer (FCB, i.e. PBS + 2% heat-inactivated FCS + 0.1% azide), cells were resuspended at $6x10^6$ / ml in FCB and stained with monoclonal antibody 2A10/11 to porcine CD163 and Alexa Fluor® 488 F(ab')2 fragment of goat anti-mouse IgG, IgM (H+L).

Orange-colored area: negative control.

White-colored area: mAb 2A10/11-stained cells.
