# Supplementary material for: Characterization of the IgA response to PRRS virus in pig oral fluids
Source: PLoS One. 2020 Mar 3;15(3):e0229065. doi: 10.1371/journal.pone.0229065 (PMC7053757; doi:10.1371/journal.pone.0229065)
Supplement: S2 Fig — Peripheral blood monocytes from three PRRS-free, SPF sows were differentiated to macrophages over 4 days. Then the non-IgG fraction of OF from test 4 (see Results section) was reacted with PRRSV strains 957 and 009/8 (5x10^6^ genome copies /ml), and medium (RPMI 1640 + 10% SFB), respectively. After 1 hour at 37°C, the virus/OF, medium/OF and medium only (control) samples were transferred onto adherent macrophages. After 1 hour at 37°C in 5% CO2, the samples were discarded, macrophages were washed twice with PBS and detached in PBS-10 mM EDTA (1 hour at 4°C), fixed in 3% formaldehyde and permeabilized in PBS-1% saponin (PBS-S). Intracellular IgA were revealed with a mAb to swine IgA (AbD Serotec, cat. MCA638) and Alexa Fluor® 488 F(ab')2 fragment of goat anti-mouse IgG, IgM (H+L). A: macrophages gated by a combination of forward and side scatter. B: gating of singlets. C: staining of intracellular IgA in macrophages of sow 3. (PDF) [file pone.0229065.s002.pdf]

# S 2 Figure

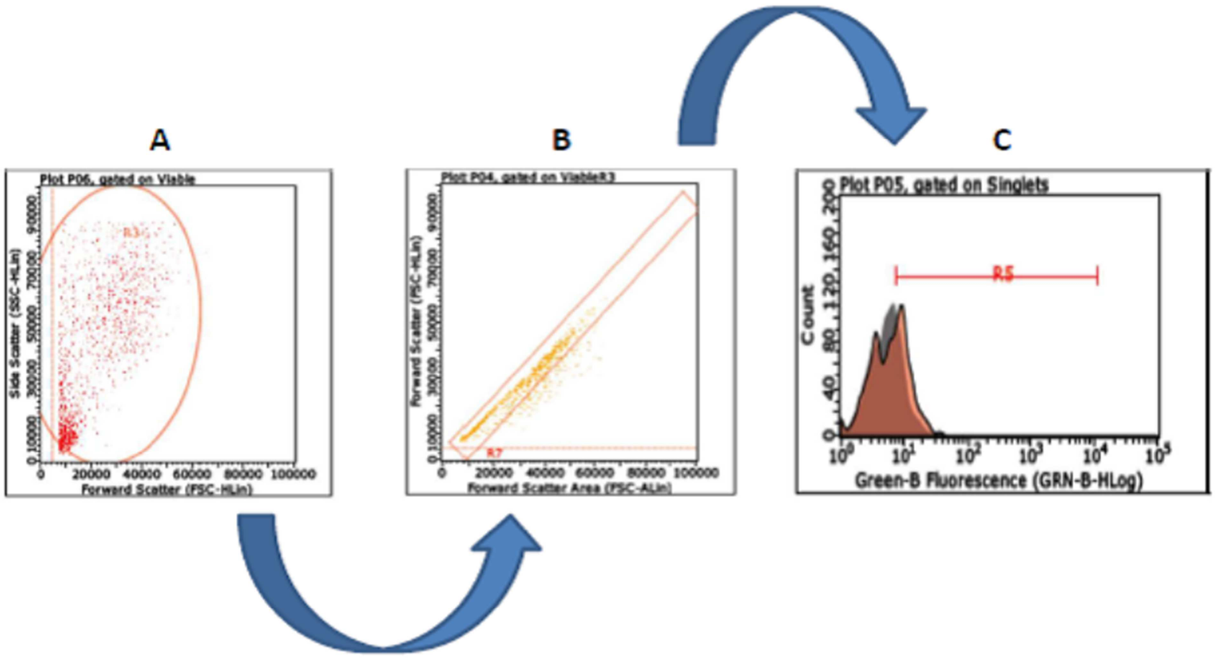


**S 2 Figure. Intracellular IgA in swine macrophages.** **P**eripheral blood monocytes from three PRRS-free, SPF sows were differentiated to macrophages over 4 days. Then the non-IgG fraction of OF from test 4 (see Results section) was reacted with PRRSV strains 957 and 009/8 ($5x10^6$ genome copies /ml), and medium (RPMI 1640 + 10% SFB), respectively. After 1 hour at 37°C, the virus/OF, medium/OF and medium only (control) samples were transferred onto adherent macrophages. After 1 hour at 37°C in 5% $CO_2$, the samples were discarded, macrophages were washed twice with PBS and detached in PBS-10mM EDTA (1 hour at 4°C), fixed in 3% formaldehyde and permeabilized in PBS-1% saponin (PBS-S). Intracellular IgA were revealed with a mAb to swine IgA (AbD Serotec, cat. MCA638) and Alexa Fluor® 488 F(ab')2 fragment of goat anti-mouse IgG, IgM (H+L). **A:** macrophages gated by a combination of forward and side scatter. **B:** gating of singlets. **C:** staining of intracellular IgA in macrophages of sow 3.
